# Overweight and obesity in pregnancy: their impact on epigenetics

**DOI:** 10.1038/s41430-021-00905-6

**Published:** 2021-07-06

**Authors:** Christoph Reichetzeder

**Affiliations:** Department of Nutritional Toxicology, Institute for Nutritional Science, University of Potsdam, Nuthetal, Germany

**Keywords:** Obesity, Epigenetics, Epigenomics, Model vertebrates, Obesity

## Abstract

Over the last few decades, the prevalence of obesity has risen to epidemic proportions worldwide. Consequently, the number of obesity in pregnancy has risen drastically. Gestational overweight and obesity are associated with impaired outcomes for mother and child. Furthermore, studies show that maternal obesity can lead to long-term consequences in the offspring, increasing the risk for obesity and cardiometabolic disease in later life. In addition to genetic mechanisms, mounting evidence demonstrates the induction of epigenetic alterations by maternal obesity, which can affect the offspring’s phenotype, thereby influencing the later risk of obesity and cardiometabolic disease. Clear evidence in this regard comes from various animal models of maternal obesity. Evidence derived from clinical studies remains limited. The current article gives an overview of pathophysiological changes associated with maternal obesity and their consequences on placental structure and function. Furthermore, a short excurse is given on epigenetic mechanisms and emerging data regarding a putative interaction between metabolism and epigenetics. Finally, a summary of important findings of animal and clinical studies investigating maternal obesity-related epigenetic effects is presented also addressing current limitations of clinical studies.

## Obesity during pregnancy

Overweight and obesity, defined as a body mass index (BMI) of 25–<30 or ≥30 kg/m^2^, respectively, have reached epidemic proportions over the last 30 years [1]. Current estimates suggest that by 2038 about 38% of the world’s population is expected to be obese [2]. In parallel with the increase in the general population, the prevalence of overweight and obesity also has increased in pregnant women [1]. Overweight and obesity are associated with a variety of pregnancy complications and are considered the most common health risks during pregnancy. These complications include gestational hypertension, preeclampsia, preterm birth, gestational diabetes mellitus (GDM), both, small and large for gestational age offspring, and a higher prevalence of stillbirth and congenital defects [3, 4]. In addition, there is growing evidence that exposure of offspring from obese mothers to an inadequate in utero environment may influence susceptibility for different non-communicable diseases in adulthood, including obesity and type 2 diabetes mellitus [5]. However, our understanding of the potential long-term effects of maternal overnutrition on the offspring and the mechanisms behind these is still limited, but a growing body of evidence highlights the importance of epigenetic modifications. Much insight in this regard comes from animal studies. In humans, however, it remains challenging to unravel the complex interaction between genetics, epigenetics, and pre- and postnatal environmental influences [6].

## Pathophysiology of obesity in pregnancy

During a healthy pregnancy, maternal physiology changes to support the growth of the fetus. This includes crucial metabolic adjustments in maternal insulin sensitivity depending on the demands of the specific pregnancy stage. In the early stages of pregnancy, there is an increase in insulin sensitivity that promotes the uptake of glucose into adipose tissue and prepares the organism for the increased energy requirements in later stages of pregnancy. However, during the progression of pregnancy, the maternal metabolism switches to a state of relative insulin resistance, resulting in a modest elevation of maternal blood glucose that is readily transported across the placenta to support fetal growth. Moreover, this low-level insulin resistance drives endogenous glucose production and utilization of fat stores, leading to further increases in blood glucose and free fatty acids [7]. To maintain adequate glucose control during pregnancy, maternal pancreatic β cells must increase insulin secretion to counteract the decrease in tissue sensitivity to insulin. In GDM, pancreatic β cells are unable to compensate for increased insulin resistance leading to the emergence of maternal glucose intolerance and GDM [8]. Obesity is associated with hyperinsulinemia and insulin resistance, which may be triggered by concomitant low-grade systemic inflammation and subclinical endotoxemia [9]. Obesity in pregnancy has been shown to significantly affect glucose metabolism leading to impaired fasting glucose reduction in early pregnancy and a considerate increase of peripheral and hepatic insulin resistance [10]. Consequently, obesity-related prepregnancy insulin resistance is associated with a strongly increased risk for GDM [1]. It was shown that changes in insulin sensitivity through the course of pregnancy are in parts related to maternal fat mass, and a marked increase in fat mass can be observed during pregnancy in both normal weight and obese women [11]. Due to the reduced effect of insulin on lipolysis, pregnancy-related insulin resistance affects lipid metabolism resulting in a several-fold increase of triglyceride and cholesterol levels late in gestation [12]. Normal weight women display net lipogenesis in early gestation (12–14 weeks) and net lipolysis in late pregnancy stages (34–36 weeks). Obese women, in contrast, show net lipolysis at all pregnancy stages. This suggests that in obese pregnancies the developing fetus is exposed to high levels of free fatty acids throughout all stages of in utero development [13]. In obese pregnancies a lipotoxic effect can be observed that promotes inflammation, endothelial dysfunction, and impairs placental invasion, which subsequently leads to alterations in placental metabolism and function [14, 15]. The supply of excess lipid and glucose to the fetus in combination with an inadequate placental function and in utero environment are thought to be relevant factors that may also increase the risk of metabolic disease in the offspring [14, 15].

## Placental changes associated with maternal obesity

In mammals, the placenta is the key element necessary for a successful pregnancy. Its various functions range from governing implantation of the early embryo and preventing its rejection by the maternal immune system, to mediating the transfer of gases, nutrients, and waste products between mother and fetus [9, 16]. The placenta is a major production site for pregnancy-related hormones that are released into the maternal circulation eliciting important functions in the adaptation of the maternal organism to and in the maintenance of pregnancy [16]. As the placenta is the main interface between mother and fetus, it is regulated by, both, fetal and maternal signals. Evidence is accumulating that the placenta acts as a sensor of the maternal-fetal environment, actively triggering adaptive responses toward intrinsic (e.g., gestational age, genetic setup) as well as extrinsic factors (e.g., nutritional variations) [16]. Furthermore, literature suggests a sexual dimorphism in adaptive placental responses [17].

Maternal overweight and obesity are associated with alterations in placental structure and function, characterized by increased inflammation and lipotoxic effects affecting nutrient transport, energy homeostasis, angiogenesis, and villous maturation [15, 18, 19]. Moreover, placental function may be compromised in maternal obesity, putatively due to impaired mitochondrial function, and increased oxidative stress [18–20]. It was shown that with increasing maternal adiposity there is a decrease in oxidative phosphorylation resulting in a lower placental cellular ATP production [19]. Similarly, another study demonstrated a reduced activity of mitochondrial respiratory complexes and increases in radical oxygen species formation in placentas of obese women, which by itself serves as a marker of impaired mitochondrial function [18]. Furthermore, it was demonstrated that maternal obesity is associated with a decreased expression of placental mitochondrial cholesterol transporter and reduced mitochondrial cholesterol concentrations. This indicates a critical modification of mitochondrial function in response to maternal obesity, which was shown to lead to a deficiency in placental endocrine output highlighted by a decreased synthesis of both progesterone and estradiol [21].

Insulin signaling plays an important role in placental growth and development that is supported by the observation of a higher trophoblastic insulin receptor expression in early gestation compared to late gestation [22]. Moreover, the area under the insulin curve determined by a glucose tolerance test at 12–14 weeks of gestation was shown to correlate with placental weight and neonatal adiposity at term, whereas this association is not found in glucose tolerance tests performed at late stages of pregnancy [23]. This suggests that the metabolic profile in early gestation dictates placental growth and development, and indicates involvement of maternal insulin in these processes [24]. It was demonstrated that maternal obesity with the accompanying insulin resistance and hyperinsulinemia is associated with a placental gene expression profile characteristic of mitochondrial dysfunction and impaired energy metabolism [25]. As outlined above, there is considerate evidence demonstrating impaired mitochondrial function in the placenta of obese women [18–21]. Compared to lean women, obese woman display an increased lipid accumulation in the placenta, which can ultimately affect the placental lipid supply to the fetus and therefore might be a cause for increased fetal adiposity in offspring of obese women [21, 25, 26]. Disturbances in placental fatty acid metabolism due to mitochondrial dysfunction, including uptake of fatty acids, decreased fatty acid oxidation, or increased esterification might be one cause for the increased lipid content found in placentas of obese women [21, 27].

## Epigenetics and maternal obesity

Environmental factors can lead to epigenetic modifications that can alter gene expression without affecting the DNA-sequence. Essential epigenetic mechanisms include histone modifications, non-coding RNAs, and DNA methylation. Epigenetic mechanisms regulate the accessibility of DNA for transcription factor complexes, the efficiency of gene transcription, and the stability of messenger RNA (mRNA). Post-translational histone modifications, including acetylation, methylation, sumoylation, and phosphorylation, impact on chromatin structure, which may influence gene transcription [28–30]. By means of RNA interference, non-coding RNAs can influence the degradation of already transcribed mRNA [29]. One of the best-studied epigenetic mechanisms is DNA methylation. DNA methylation is defined as the addition of a methyl group to cytosine and is carried out by DNA methyltransferases, requiring the cofactor S-adenosylmethionine. Cytosine methylation can be mitotically inherited; thus, DNA methylation is considered a mechanism of somatic inheritance. In mammals, DNA methylation usually takes place at cytosines in a cytosine-guanine context referred to as “CpG” (the “p” stands for the intervening phosphate group), converting cytosine to 5-methylcytosine (5mC). CpG rich regions in the DNA are scattered unevenly across the genome and are generally referred to as “CpG islands.” CpG islands are usually found in higher numbers in promoter regions of genes. CpG methylation affects the expression of genes by binding to methylation-sensitive DNA binding proteins and by the interaction with different histone tail modifications regulating DNA accessibility [13]. In general, hypomethylation of promoter regions leads to an increased expression of a respective gene, whereas hypermethylation results in transcriptional repression [30]. DNA can also be actively demethylated by members of the ten eleven translocation (TET) dioxygenase enzyme family. The process of active demethylation encompasses several steps, starting with the conversion of 5mC to 5-hydroxymethylcytosine (5hmC). Next, 5hmC is converted to 5-formylcytosine (5fmC) and ultimately to 5-carboxylcytosine (5CmC). Both 5fmC and 5CmC can be processed by thymine-DNA glycosylase, creating an abasic site that is converted back to cytosine by base excision repair. It has been implicated that active demethylation of the genome has a regulatory function responsible for fine-tuning methylation marks [31]. Furthermore, evidence is accumulating that 5hmC is not just a passive intermediate during active demethylation but elicits unique epigenetic functions [32]. Obesity is known to be associated with altered patterns of placental gene expression and epigenetic changes including histone modifications, DNA methylation and hydroxymethylation, and microRNA expression [33–35].

**Fig. 1:**
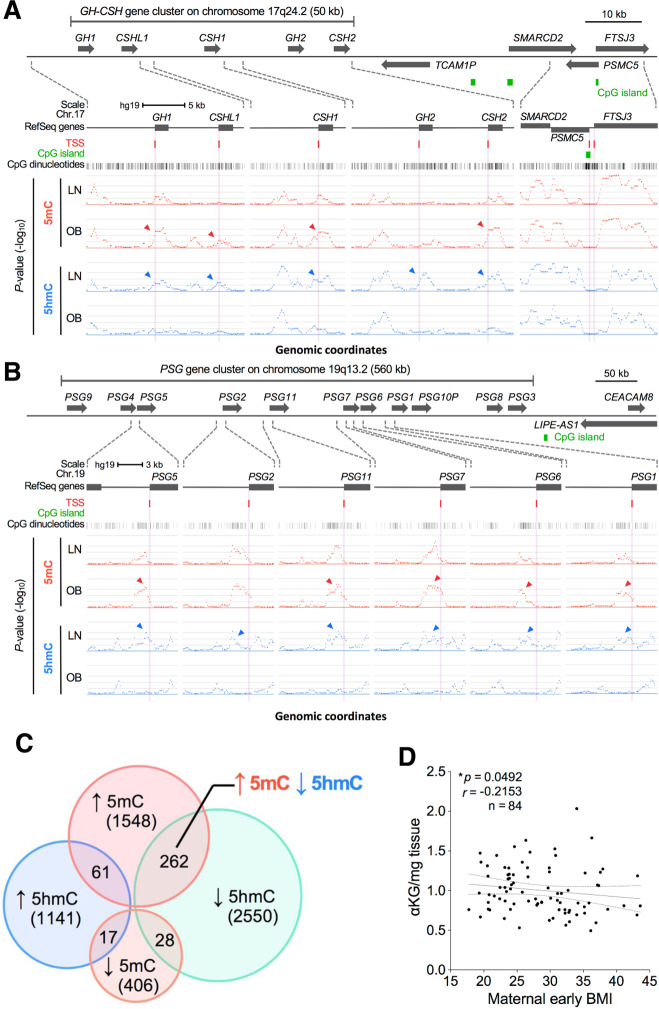
Alterations in the placental methylome with maternal obesity and evidence for metabolic regulation. **A**, **B** Increased DNA methylation and reciprocally decreased DNA hydroxymethylation at pregnancy-associated gene clusters in placentas of obese compared to lean mothers. **A** GH-CSH gene cluster (located on human chromosome 17q24) analysis demonstrating a reciprocal increase in 5mC and decrease in 5hmC. Differential DNA methylation (5mC, red) and hydroxymethylation (5hmC, blue) were observed at the human growth hormone-chorionic somatomammotropin (hGH/hCS/hPL) gene cluster between epigenomes of lean (LN) and obese (OB) mothers. In contrast, a strong resemblance in respective 5mC and 5hmC profiles was evident outside the GH-CSH gene cluster as shown in the rightmost panel. **B** PSG gene cluster (located on human chromosome 19q13) analysis demonstrating a reciprocal increase in 5mC and decrease in 5hmC. Genomic features are shown as custom tracks in the UCSC genome browser. TSS transcriptional start sites. Arrowheads in the figures indicate marked differences in 5mC and 5hmC distributions. Distribution of genomic 5mC and 5hmC was performed employing methylated and hydroxymethylated DNA immunoprecipitation (MeDip; hMeDIP). **C** Venn diagram illustrating overlaps of differentially methylated and hydroxymethylated loci. Extended analysis of MeDIP and hMeDIP data sets leads to the identification of a total of 5645 differentially methylated or hydroxymethylated genes across the entire genome (1548 loci with increased 5mC, 406 loci with decreased 5mC, 1141 loci with increased 5hmC, and 2550 loci with decreased 5hmC in obese vs. lean pregnancies). **D** Correlation between maternal early pregnancy BMI and placental α-ketoglutarate levels determined by HPLC-ESI-MS. The relationship between the continuous variables was evaluated by Spearman correlation analysis. Reprinted from [33] published under a CC BY 4.0 license.

**Fig. 2:**
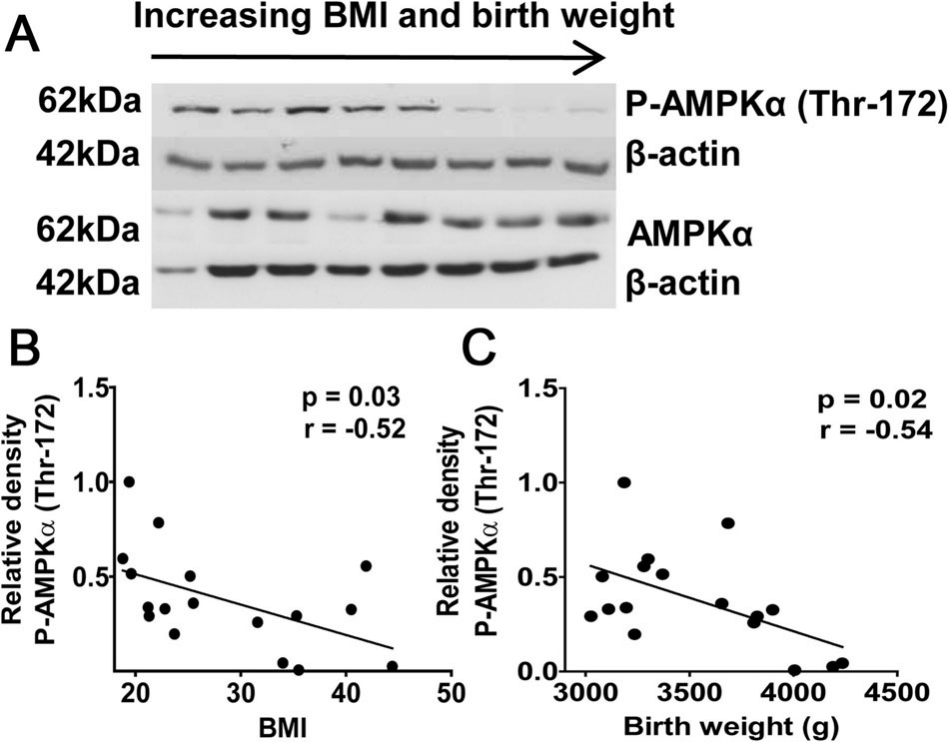
Placental AMPK signalling in relation to maternal BMI and offspring birth weight. Among other findings, an inverse correlation between AMPK phosphorylation and birth weight was demonstrated. This is an intriguing finding also from an epigenetic perspective, given the relationship between AMPK and TET enzymes, as outlined in chapter 1.4. **A** Representative western blots for total and phosphorylated AMPKα (Thr-172) in placental homogenates from pregnancies with varying BMI and birth weights. No significant correlation between BMI or birth weight and total AMPKα was observed. **B** Relationship between BMI and phosphorylated placental AMPKα. **C** Relationship between birth weight and phosphorylated placental AMPKα. *N* = 17; *r* = Pearson’s correlation coefficient. Reprinted from [39] by permission of Oxford University Press. The figure is notincluded in the current article’s Creative Commons license.

The underlying mechanisms that trigger obesity-related epigenetic changes are still incompletely understood. However, recent findings suggest a possible link between metabolism and epigenetics. Comparing placental tissue of obese versus lean pregnancies, Mitsuya et al. investigated placental DNA methylation and hydroxymethylation on a genomic scale. They were able to show a partial but significant overlap of genes that experienced an increase in DNA methylation and a reciprocal decrease in DNA hydroxymethylation with increased maternal obesity, suggesting a possible decrease in the conversion efficiency of methylation to hydroxymethylation, which is governed by TET dioxygenases (Fig. 1). TET dioxygenases require α-ketoglutarate (αKG) as an essential cofactor that is formed from isocitrate in the mitochondrial tricarboxylic acid cycle by isocitrate dehydrogenase. Measuring αKG levels in placental tissue, the authors were able to demonstrate a modest but significant negative correlation between αKG levels and maternal early BMI (Fig. 1) [33]. Previously, evidence from metabolomic studies demonstrated a decrease in αKG levels in obese individuals and increases in αKG following weight loss [36, 37]. Since α-KG synthesis occurs largely in mitochondria, a potential connection between decreases in αKG and obesity might be disturbances in mitochondrial function and homeostasis, a common finding in placentas from obese women [18, 19, 21, 36]. Support for this notion comes from an animal study, which showed a high-fat diet-associated α-KG decrease in cardiac tissue that was attributable to impaired mitochondrial function [38]. Also other evidence points toward a connection between metabolism and epigenetics. Current literature suggests that dysregulation of adenosine monophosphate-activated protein kinase (AMPK) and mammalian target of rapamycin (mTOR) homeostasis is a relevant factor in gestational obesity and associated fetal overgrowth (Fig. 2) [39]. AMPK activation occurs in response to falling energy levels indicated by high AMP and low ATP concentrations. In situations of low cellular ATP levels, activation of AMPK leads to an inhibition of mTOR signaling, thereby preserving cellular energy during such low energy states [40]. Wu et al. demonstrated that activation of AMPK by falling cellular energy levels phosphorylates and stabilizes TET2. Under conditions that inhibit AMPK signaling, such as high glucose, AMPK was no longer activated, resulting in loss of TET2 phosphorylation and stability, decreased TET2 levels, and reduced nuclear levels of DNA hydroxymethylation [41]. Concerning obesity in pregnancy, it has recently been shown that prepregnancy BMI shows a negative correlation with AMPK activation in the placenta [42]. A study investigating the effects of obesity on the placenta in sows demonstrated an association between obesity and a lipotoxic placental milieu that was characterized by a decreased AMPK activation [43]. In a similar finding, a negative correlation between maternal prepregnancy BMI and AMPK expression was demonstrated for fetal cord blood, also highlighting that maternal BMI can act as an important factor in programming protein expression at birth in the offspring [44]. Taken together, evidence is accumulating that points toward a mechanistic connection between the metabolic state of a cell and its epigenome, which might have implications in obesity-related epigenetic modifications. Future studies are still needed to better characterize this putative mechanistic link between metabolism and epigenetics and also to disentangle the associated “chicken or egg” causal dilemma.

## Epigenetic alterations in the offspring of obese mothers

It is well known that metabolic diseases such as obesity have a multifactorial etiology, with genetic and environmental factors contributing to disease development. Regardless of crucial environmental factors, such as the combination of an energy-dense diet and a sedentary lifestyle with a decreased energy expenditure, clear evidence highlighting a strong genetic basis of obesity exists [45]. The predicted genetic contribution to the obesity-related anthropometric trait of BMI was initially estimated from twin studies to range between 40 and 70% [46]. However, alternative statistical methods have suggested that these estimates may have been too high, with the genetic contribution rather ranging between 30 and 40% [46, 47]. More recently, whole-genome association studies (GWAS) have emerged as a tool for a non-hypothesis-driven identification of novel genes and loci contributing to obesity [45]. GWAS revealed important novel insights into the genetics of obesity; however, the majority of studies have estimated the overall contribution of identified GWAS single nucleotide polymorphisms (SNPs) to the observed variance ranges between 5 and 15%. It was suggested that gene-gene and gene-environment effects and lack of rare variant coverage might be factors responsible for the underestimation of identified trait-linked loci. Moreover, the majority of disease and trait-linked SNPs were demonstrated to be located in non-coding portions of the genome. Thus, GWAS results often indicate disease-associated variants that modulate risk by altering functional DNA elements that regulate gene expression. However, identifying the corresponding target genes remains challenging [46, 48]. In summary, there is evidence for a rather strong genetic component in the development of obesity, but the heritability of obesity cannot entirely be attributed to genetic variation [45, 46, 49].

Next to genetic mechanisms, recent evidence indicates an involvement of epigenetic mechanisms in the intergenerational effects of maternal obesity. Seminal work by Barker et al. increased the popularity of a hypothesis previously put forward by other scientists—the so-called fetal programming hypothesis [50, 51]. This hypothesis states that certain environmental influences during embryonic, fetal, and also neonatal development can permanently affect the phenotype of an organism. The fact that epigenetic mechanisms (e.g., DNA methylation, histone modifications, expression of non-coding RNAs) can be influenced by environmental factors, particularly in early life, serves as the basis for the fetal programming hypothesis, which posits that specific intrauterine environments can lead to altered epigenomes and ultimately different phenotypes of a whole organism [51, 52].

**Fig. 3:**
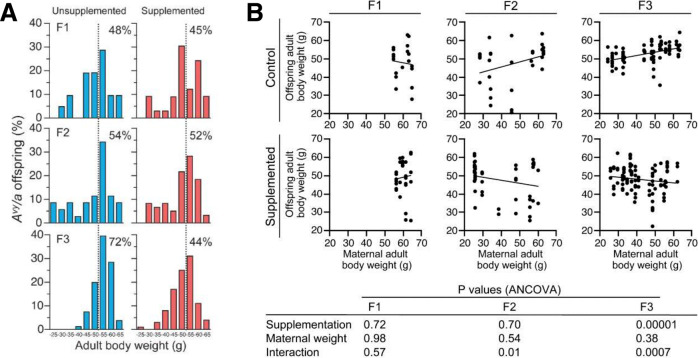
Methyl supplementation prevents transgenerational increase in adult body weight. In the study by Waterland et al. Agouti viable yellow (Avy) mice were employed to test the hypothesis that maternal obesity can induce a transgenerational amplification of obesity. The A^vy^ allele was passed through three generations of A^vy^/a female mice and the cumulative effects on coat color and body weight were investigated. A potential mediation of transgenerational effects on body weight by DNA methylation was studied by analyzing two separate but contemporaneous populations of mice, one fed a standard diet and the other a methyl-supplemented diet capable of inducing DNA hypermethylation during development. The authors demonstrated the prevention of transgenerational increases in adult body weight by methyl supplementation. **A** Distribution of adult (P180) body weight of A^vy^/a offspring by generation and group; *n* of the unsupplemented groups: 29 (F1), 36 (F2), and 88 (F3); *n* of the supplemented groups: 33 (F1), 62 (F2) and 117 (F3). The percentages in each panel indicate the proportion of offspring above 50 g (dotted line). Body weight was relatively constant in the supplemented group but increased transgenerationally in the unsupplemented group (*P* = 0.000006). **B** Methyl supplementation changed the association between maternal and offspring adult body weight. Adult body weight was measured at P180 for all dams and A^vy^ offspring in the study. In both the F2 and F3 generations, maternal adult body weight predicted offspring adult body weight in the unsupplemented group only. The *P* values of the analysis of covariance are provided. Reprinted by permission from Springer Nature, International Journal of Obesity, Methyl donor supplementation prevents transgenerational amplification of obesity, [57]. The figure is not included in the current article’s Creative Commons license.

Although many initial fetal programming studies were focused on maternal undernutrition, it was soon demonstrated in animal and clinical studies that maternal overnutrition can also lead to epigenetically mediated alterations in different physiological homeostatic regulatory systems and is associated with increases in the cardiometabolic risk in the offspring [51, 53]. Clear evidence that the maternal diet can epigenetically predispose offspring phenotype to metabolic disease in adulthood comes from studies investigating the Agouti yellow mouse (A^vy^/a). Agouti yellow mice boast a metastable epiallele that triggers a specific phenotype. The term epiallele is used for alleles of the same genes that only differ in their DNA methylation status. Moreover, metastable epialleles refer to epialleles in which the degree of DNA methylation can be altered by environmental factors [54]. Consequently, the methylation status can influence the expression of a metastable epiallele. Differences in promoter methylation of such metastable epialleles can lead to different phenotypes of genetically identical individuals [54, 55]. The Agouti gene encodes for a paracrine signaling molecule that leads to yellow hair follicle pigmentation and has an antagonistic effect on hypothalamic melanocortin 4-mediated satiety signaling. Therefore, A^vy^/a mice have yellow fur and develop obesity via hyperphagia. Mice that are carriers of a silent allele, in contrast, have Agouti colored coats and display a metabolically inconspicuous phenotype [56]. Using A^vy^ mice, an important study by Waterland et al. was able to demonstrate that maternal obesity accumulates over several generations, resulting in a shift toward higher adult body weight in the investigated population. Furthermore, it was shown that the transgenerational effects on body weight could be inhibited by providing a methyl-rich diet that induces DNA hypermethylation during development, indicating that the observed effects were mediated by epigenetic alterations (Fig. 3) [56, 57]. Further studies by Li et al. demonstrated that the offspring of obese yellow A^vy^/a dams display a latent metabolic disease predisposition, which could be unmasked by providing a high-fat diet. Genome-wide profiling of DNA methylation demonstrated changes in the methylome with a significant clustering in developmental ontologies. It was concluded that genes necessary for developmental processes appear to be more susceptible to environmental challenges compared to other groups of genes and that the observed epigenetic alterations could likely underlie the offspring’s metabolic phenotype resulting from exposure to maternal obesity [56, 58, 59].

**Fig. 4:**
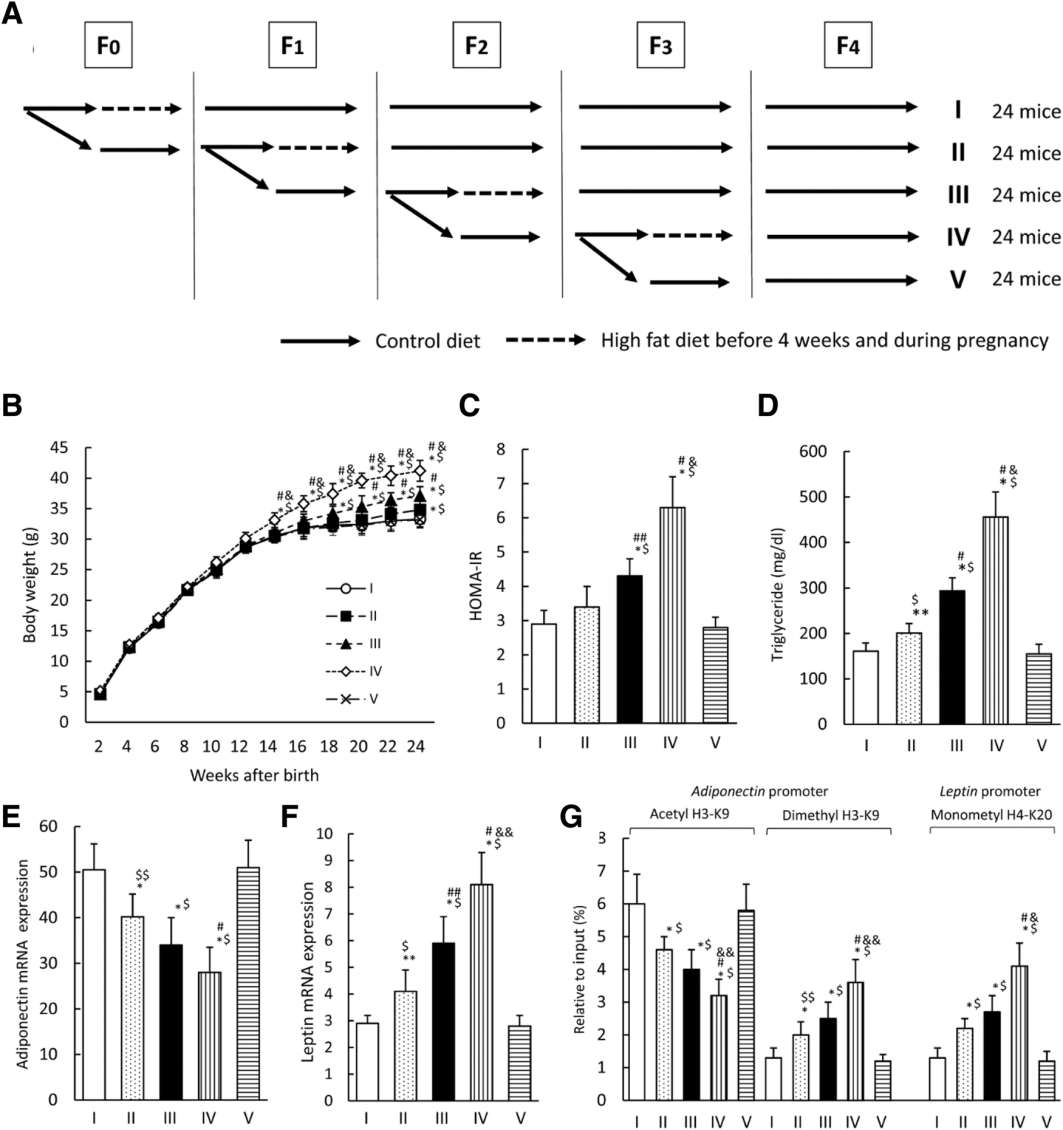
Transgenerational effects on the metabolic phenotype of in utero high-fat diet exposure. The study by Masuyama et al. investigated transgenerational effects on the metabolic phenotype of in utero high-fat diet exposure in ICR mice. To this end, in utero high-fat diet-exposed female offspring were analyzed for several generations and it was also studied if exposure to a standard control diet can diminish or abolish transgenerational effects of the high-fat diet. **A** Scheme of the experimental setup. Following groups were generated: female offspring from dams fed with a control diet before and during pregnancy after exposure to a high-fat diet in utero in 3 (I), 2 (II), and 1 (III) generations; female offspring from dams fed a high-fat diet (IV); female offspring from dams fed a control diet in all generations (V). **B**–**F** The effects of a control diet before and during pregnancy on offspring after high-fat diet exposure in utero. **B** Body weight development, **C** HOMA-IR, **D** serum triglyceride levels, **E** mRNA expression of adiponectin, **F** mRNA expression of leptin, **G** histone modifications of H3K9 and H4K20 in the promoter regions of adiponectin and leptin in the adipose tissue of offspring in groups I–V. Results are given as mean ± SD; *n* = 12 females per group; *P < 0.01 and **P < 0.05 versus group V; ^$^*P* < 0.01 and ^$$^*P* < 0.05 versus group I; ^#^*P* < 0.01 and ^##^*P* < 0.05 versus group II; ^&^*P* < 0.01 versus group III. Data are presented as mean ± SD. Reprinted from [65] by permission of Oxford University Press. The figure is not included in the current article’s Creative Commons license.

Different recent rodent studies have investigated the influence of gestational maternal high-fat diets on epigenetic alterations in adipose tissue, and the liver of the offspring [60–62]. A study by Masuyama and Hiramatsu demonstrated that reduced adiponectin and increased leptin mRNA expression in adipose tissue in offspring of gestational high-fat diet dams were caused by increased acetylation and decreased methylation of histone H3K9 in the adiponectin promoter and increased methylation of histone H4K20 in the leptin gene. Furthermore, an association was found between the observed epigenetic modifications and hypertension, insulin resistance, and hyperlipidemia in the offspring [60]. Similarly, other studies demonstrated the involvement of histone modifications affecting gene expression of phenotype relevant genes in the offspring of high-fat diet dams [61, 63]. Regarding the transgenerational impact of obesity, results of a study by Li et al. implicated an accumulation of epigenetic modifications including histone methylation that contributed to increased lipogenesis over several generations, making F_2_ offspring derived from both grand-maternal and maternal obesity extremely prone to developing obesity [64]. Supporting this finding, Masuyama et al. observed that the effects of maternal high-fat feeding were reversed in the offspring only after feeding a standard diet for three consecutive generations (Fig. 4) [65].

Investigating other epigenetic mechanisms beyond histone modifications, several studies observed differences in DNA methylation in offspring from dams receiving high-fat diets throughout gestation. Exposure to maternal obesity during development is associated with increased white adipose tissue in the progeny [28]. A study investigating potential epigenetic mechanisms underlying this observation demonstrated alterations in global DNA methylation of CpG sites and islands in white adipose tissue associated with an upregulation of lipogenic pathways. Yang et al. reported that maternal obesity reduces DNA methylation in the zinc finger protein 423 (*zfp423*) promoter, a gene that functions as a key regulator of progenitor cell commitment towards preadipocyte differentiation. Hypomethylation of the *zfp423* promoter was correlated with increased *zfp423* expression and progenitor adipogenesis in fetal mouse tissue [66]. Premature adipogenic differentiation in adipose tissue of the offspring negatively affects the pool of progenitor cells, limiting adipose tissue expandability, especially when exposed to a high‐energy diet. The inability to expand adipose tissue results in adipocyte hypertrophy, an important cause of hypoxia and inflammation [67]. Taken together, epigenetic changes, more precisely alterations in DNA methylation, in key genes regulating adipogenesis and the maintenance of an adipocyte progenitor cell pool appear to be a relevant mechanism in shaping the offspring’s phenotype by maternal obesity [28].

In addition to histone modifications and DNA methylation, the expression of micoRNAs was also demonstrated to be involved in affecting adipose tissue metabolism in offspring of obese dams [68, 69]. Fernandez-Twinn et al. demonstrated that lean offspring of obese dams display an increased adipose tissue expression of miR-126, which was accompanied by a reduced expression of the insulin signaling protein IRS-1, one of the targets of miR-126. As it was possible to maintain the adipocyte phenotype in in vitro expanded and differentiated epididymal adipose tissue precursor cells from offspring of obese dams, the authors hypothesized that the underlying mechanism acts in a cell-autonomous manner that may drive insulin resistance in later life [69].

**Fig. 5:**
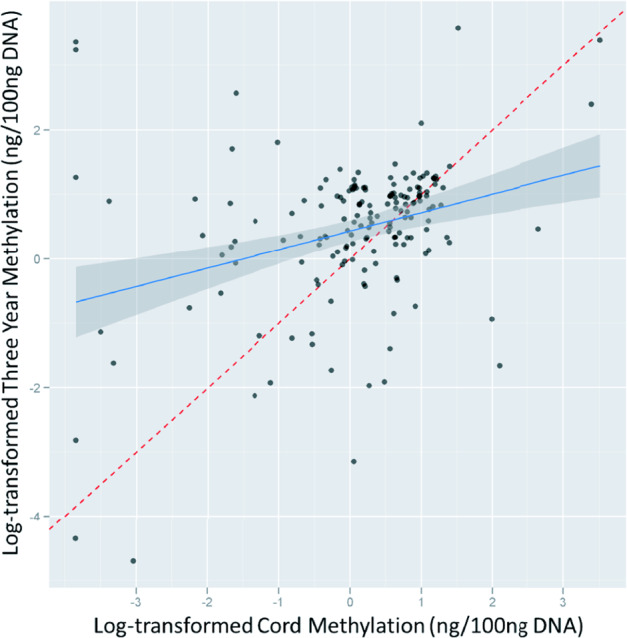
Association between cord and 3-year DNA methylation. Herbstman et al. investigated if levels of global DNA methylation persist within an individual from birth to age 3. Global DNA methylation was measured in the same children (*n* = 165) at birth (cord blood) and again at 3 years of age in isolated total blood leukocytes using an immunoassay. Cord and 3-year DNA methylation were moderately but significantly correlated (Pearson’s *R* = 0.41 (*P* < 0.001), Spearman *R* = 0.30 (*P* < 0.001)). Reprinted from [71] published under a Creative Commons Attribution license.

**Fig. 6:**
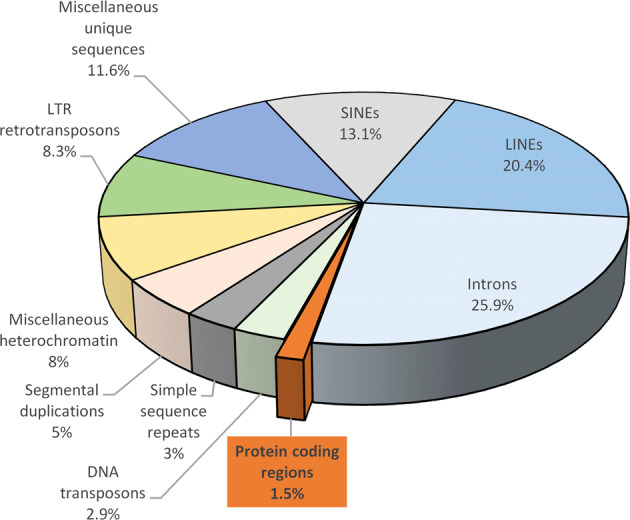
Overview of different components that make up the human genome. Only about 1.5% of the human genome actually consists of protein coding regions, whereas the large majority is composed of non-coding sequences, such as introns and transposable elements (mostly devoid of function) including long interspersed nuclear elements (LINE), short interspersed nuclear elements (SINE), long terminal repeat (LTR) retrotransposons. Re-drawn figure by permission from Springer Nature [75]. The figure is not included in the current article’s Creative Commons license.

Several clinical studies have investigated associations between maternal obesity and epigenetic changes in offspring. A major limitation in the setting of clinical studies investigating maternal obesity mediated epigenetic effects on the offspring is the fact that it is not possible to investigate samples of relevant target organs. Therefore, tissues that allow minimally invasive sampling are usually used. These include blood samples, to investigate circulating factors such as non-coding/micro RNAs, isolation of leukocyte DNA from umbilical cord blood, and tissue samples of the placenta. Commonly, DNA methylation of leukocyte DNA is evaluated as surrogate parameter for epigenetic changes in the whole organism. The true significance of changes in leukocyte DNA methylation as a surrogate marker for organismal epigenetic changes is not yet fully understood, but current literature suggests that this parameter may serve as a useful risk biomarker [5, 70]. It was demonstrated that changes in DNA methylation in leukocytes are stable and that associations between maternal BMI during pregnancy and global DNA methylation at the age of 3 years still exist (Fig. 5) [71]. Another issue of currently available data from clinical studies investigating epigenetically mediated effects of maternal obesity is the lack of replication of obtained results in suitably designed replication cohorts [5]. This problem is further aggravated, especially in DNA methylation studies, by a multitude of different methodological approaches in the analysis and quantification of DNA methylation. In this regard, a major distinction can be made between assays that determine gene-specific, genome-wide, or global DNA methylation. In the study of gene-specific and genome-wide DNA methylation, DNA methylation is mainly studied in promoter regions of genes that are rich in CpG sites. In commonly used array approaches only a fraction of genomic CpGs are covered and measurement is to some degree biased toward the measurement of promoter methylation, neglecting other regions and functions of DNA methylation [72–74]. Protein encoding genes account for only about 1.5% of the total genome sequence, with the remaining bulk consisting of introns, repetitive elements, and other non-coding sequences (Fig. 6) [75]. As recent research has demonstrated important functions of DNA methylation in such non-coding genomic regions, their neglect of analysis in currently available array approaches confers a limitation [72–74]. Even newer variants of genome-wide array approaches like the Illumina MethylationEPIC bead chip remain to some extent limited due to the exclusion of regions of potentially meaningful biological variation [76]. Other approaches, such as different sequencing methods, exis for a detailed, site-specific investigation of global DNA methylation. However, such methods currently have limited value in the analysis of DNA methylation in large study settings due to high costs and time-consuming protocols [77]. Next to array and sequencing-driven approaches also various methods exist that measure the amount of methylated cytosines in a global fashion. Commonly used approaches in this regard estimate global methylation by measuring DNA methylation of surrogates such as LINE-1 or Alu elements or by employing mass spectrometry for the quantification of global DNA methylation and hydroxymethylation. Until now, the significance of measuring global DNA methylation is not well understood, but several studies have shown that this parameter can be used as a crude biomarker to assess environmental influences and alterations in global DNA methylation have been demonstrated in obesity [78–80].

**Fig. 7:**
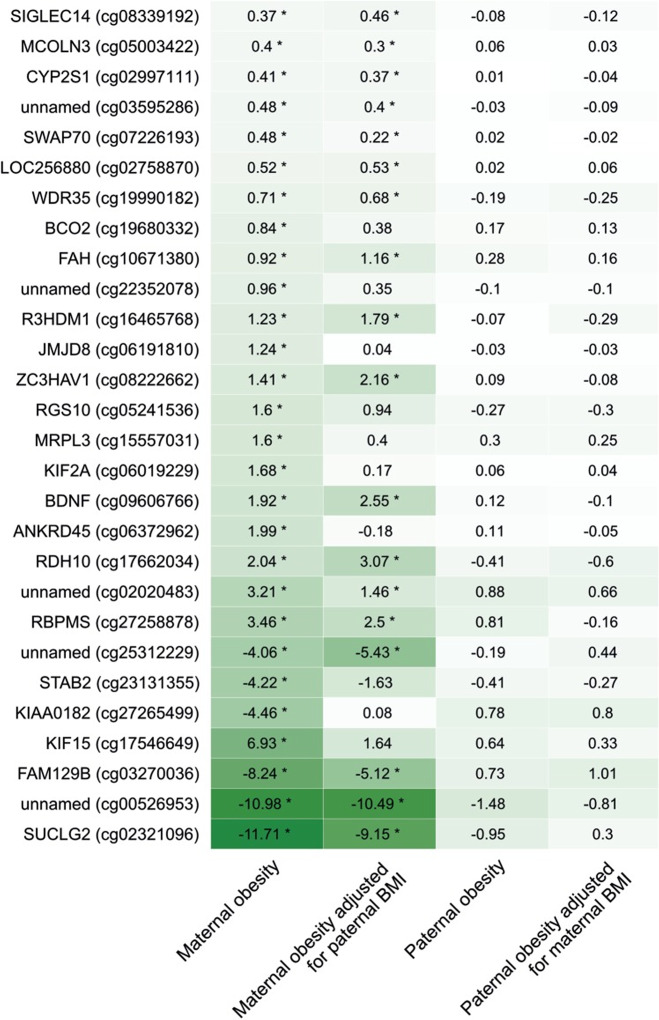
Associations between maternal or paternal obesity and offspring cord blood DNA methylation compared with offspring of normal weight mothers/fathers. Sharp et al. investigated genome-wide DNA methylation in relation to maternal and offspring adiposity in a cohort of 1018 mother–child pairs using multivariable linear regression models. The figure shows associations between maternal or paternal obesity and cord blood DNA methylation in the offspring. The numbers indicate mean differences (given in %) in offspring cord blood DNA methylation that were found comparing offspring of obese mothers/fathers with offspring of normal weight mothers/fathers. A larger effect size is indicated by darker shading (regardless of direction). The calculated models were adjusted for bisulfite conversion batch, and paternal/maternal continuous BMI were indicated, but no other covariates were used (obese mothers, *n* = 40; normal weight mothers, *n* = 665; obese fathers, *n* = 53; normal weight fathers, *n* = 372). Asterisk in the figure indicate associations with an FDR-adjusted *P* < 0.05. Reprinted from [82] published under a Creative Commons CC BY license.

Employing array-based genome-wide DNA methylation analysis, several studies demonstrated associations between maternal BMI and offspring cord blood DNA methylation [81, 82]. Investigating a large cohort of mother–child pairs, Sharp et al. showed that the impact of maternal obesity on genome-wide methylation was stronger compared to the impact of paternal obesity, suggesting an intrauterine mechanism (Fig. 7) [82]. In another study, Sharp et al. performed a meta-analysis of the association between prepregnancy maternal BMI and methylation at over 450,000 sites in cord blood DNA using data from 19 cohorts including a total of 9340 mother–newborn pairs [83]. To infer causality, an attempt was made to incorporate genetic information in the analyses and to compare the effects of maternal and paternal BMI. In additional cohorts including 1817 mother–child pairs with available blood samples, the association between maternal BMI at the beginning of pregnancy and whole blood DNA methylation in adolescent offspring was meta-analyzed. Results of the study revealed an association between maternal BMI and cord blood DNA methylation variation at 9044 sites that was strongly reduced to 104 CpG sites after adjusting for estimated cell proportions. Comparing the results from cord blood to adolescent blood samples, the same direction associations were observed in 72 CpG sites indicating a potential persistence of signals. However, evidence supporting a causal, maternal BMI mediated intrauterine effect on cord blood methylation was demonstrated at just eight CpG sites. The authors concluded that these observed minute effects might be more likely due to genetic or lifestyle factors than due to a causal intrauterine mechanism. Results of this study demonstrate the need for large-scale collaborative study settings and the employment of causal inference techniques in epigenetic epidemiologic studies [83]. A very recent study by Martin et al. that determined genome-wide CpG methylation in cord blood leukocytes using the Illumina HumanMethylation450k BeadChip in 361 mother–child pairs focused on investigating sex-specific effects of maternal obesity on the offspring [84]. It was shown that maternal obesity before pregnancy was associated with the methylation of 876 CpGs in female offspring and only 293 CpGs in male offspring. However, in both female and male offspring of obese mothers, hypermethylation in CpG sites of the TAPBP gene was observed [84]. Interestingly, hypermethylation of TAPBP gene CpG sites measured in neonatal blood spot samples of 438 children also using the Illumina HumanMethylation450k BeadChip was previously demonstrated to be associated with an increased cardiometabolic risk in children in another study [85]. Thus, methylation of the TAPBP gene may be considered as an epigenetic mechanism by which maternal obesity increases the cardiometabolic risk of the offspring. However, the analysis of a replication cohort performed by Martin et al. in 751 mother–child pairs was not able to replicate the initial findings. The authors attributed the lack of replication to vastly different levels of obesity and differences in the ethnic composition of the two investigated cohorts [84].

Taken together, there is ample evidence existing that demonstrates epigenetically mediated effects of maternal obesity during pregnancy on weight development and cardiometabolic risk factors in the offspring. Currently, clear evidence in this regard still predominantly stems from animal studies. To substantiate existing evidence of clinical studies, more large-scale clinical trials are necessary. As highlighted by a few existing studies, potential future studies should employ combinations of genome-wide and epigenome-wide association studies, to better delineate what contribution the respective mechanism makes.
